# Effect of switching to erenumab in non-responders to a CGRP ligand antibody treatment in migraine: A real-world cohort study

**DOI:** 10.3389/fneur.2023.1154420

**Published:** 2023-03-22

**Authors:** Lucas Hendrik Overeem, Kristin Sophie Lange, Mira Pauline Fitzek, Anke Siebert, Maureen Steinicke, Paul Triller, Ja Bin Hong, Uwe Reuter, Bianca Raffaelli

**Affiliations:** ^1^Department of Neurology, Charité – Universitätsmedizin Berlin, Berlin, Germany; ^2^Doctoral Program, International Graduate Program Medical Neurosciences, Humboldt Graduate School, Berlin, Germany; ^3^Universitätsmedizin Greifswald, Greifswald, Germany; ^4^Clinician Scientist Program, Berlin Institute of Health (BIH), Berlin, Germany

**Keywords:** migraine, preventive treatment, monoclonal antibodies, Calcitonin Gene-Related Peptide, switch, refractory migraine

## Abstract

**Background:**

Therapeutic options for migraine prevention in non-responders to monoclonal antibodies (mAbs) targeting Calcitonin Gene-Related Peptide (CGRP) and its receptor are often limited. Real-world data have shown that non-responders to the CGRP-receptor mAb erenumab may benefit from switching to a CGRP ligand mAb. However, it remains unclear whether, vice versa, erenumab is effective in non-responders to CGRP ligand mAbs. In this study, we aim to assess the efficacy of erenumab in patients who have previously failed a CGRP ligand mAb.

**Methods:**

This monocentric retrospective cohort study included patients with episodic or chronic migraine in whom a non-response (< 30% reduction of monthly headache days during month 3 of treatment compared to baseline) to the CGRP ligand mAbs galcanezumab or fremanezumab led to a switch to erenumab, and who had received at least 3 administrations of erenumab. Monthly headache days were retrieved from headache diaries to assess the ≥30% responder rates and the absolute reduction of monthly headache days at 3 and 6 months of treatment with erenumab in this cohort.

**Results:**

From May 2019 to July 2022, we identified 20 patients who completed 3 months of treatment with erenumab after non-response to a CGRP ligand mAb. Fourteen patients continued treatment for ≥6 months. The ≥30% responder rate was 35% at 3 months, and 45% at 6 months of treatment with erenumab, respectively. Monthly headache days were reduced from 18.6 ± 5.9 during baseline by 4.1 ± 3.1 days during month 3, and by 7.0 ± 4.8 days during month 6 compared to the month before treatment with erenumab (*p* < 0.001, respectively). Responders and non-responders to erenumab did not differ with respect to demographic or headache characteristics.

**Conclusion:**

Switching to erenumab in non-responders to a CGRP ligand mAb might be beneficial in a subgroup of resistant patients, with increasing responder rates after 6 months of treatment. Larger prospective studies should aim to predict which subgroup of patients benefit from a switch.

## 1. Introduction

Monoclonal antibodies (mAbs) targeting Calcitonin Gene-Related Peptide (CGRP) and its receptor are effective, well-tolerated, and safe for the preventive treatment of patients with both episodic migraine (EM) and chronic migraine (CM) (1–3).

Randomized controlled trials (RCT) have described a reduction of monthly migraine days (MMD) of ≥50% at 3 months in up to 62% of patients with EM, and in up to 41% of patients with CM treated with CGRP ligand or receptor mAbs, respectively (4–11). Evidence from real-world studies (RWS) indicates even higher response rates, with a ≥50% response rate of up to 70% to erenumab in a population of patients with EM and CM having failed ≥2 prior preventive treatments (12).

However, in both RCT as well as RWS, a variable proportion of patients did not respond to treatment with CGRP mAbs. In real-world settings, these non-responders have often already failed numerous previous preventive treatment attempts, since this is a prerequisite for reimbursement of CGRP mAbs in many countries (12–14). At the time of the present study, German reimbursement regulations required failure of ≥4 (EM) and ≥5 (CM) first-line migraine preventive medications due to either insufficient efficacy, adverse effects, or contraindications before initiation of a CGRP mAb therapy (15). These first-line preventatives include beta-blockers (metoprolol or propranolol), amitriptyline, topiramate, flunarizine, and additionally OnabotulinumtoxinA for CM (15). Switching from a CGRP ligand mAb to a CGRP receptor mAb and vice versa is a possibility in the absence of alternatives. There are no direct comparisons of the efficacy of different CGRP mAbs or recommendations on which of the available CGRP mAbs to use in a given patient.

Evidence on the efficacy of switching between CGRP mAbs is scarce. While no RCT has addressed this topic, two real-world studies and one case series have shown a ≥30% response in 32% (8/25), 100% (3/3), and 53% (8/15) of patients who switched to a CGRP ligand mAb after non-response to erenumab, respectively (16–18). In a preliminary subgroup analysis of the FINESSE study, switching to fremanezumab in patients with EM or CM and a prior ineffective treatment with either galcanezumab or erenumab resulted in a ≥50% response in 32% (18/57) of patients (19). The efficacy of a switch from a CGRP ligand mAb to erenumab due to partial or insufficient efficacy has been investigated in one real-world study and two case series, reporting a heterogeneously defined efficacy at 3 months in 63.6% (14/22), 100% (2/2), and 50% (2/4) of patients (20–22).

To expand the evidence on this clinically relevant question, we aimed to assess treatment efficacy of erenumab in patients in whom treatment with a CGRP ligand mAb had failed to reduce MHD by ≥30%, corresponding to a clinically meaningful response (23–25).

## 2. Methods

### 2.1. Study design and patient selection

This is a monocentric retrospective longitudinal cohort study conducted at the tertiary Headache Center, Charité – Universitätsmedizin Berlin, Germany. From our treatment logs, we systematically screened all patients with EM or CM who had received prophylactic treatment with a CGRP ligand mAb between May 2019 and July 2022. Patients were eligible for our study if they (i) received a first CGRP-targeting treatment with galcanezumab (240 mg loading dose followed by 120 mg monthly) or fremanezumab (225 mg monthly) for ≥ 3 months, (ii) were non-responders, i.e., reported a reduction of MHD by < 30% during month 3 compared to baseline, (iii) switched to erenumab (70 mg or 140 mg monthly, as decided by the treating physician) as second CGRP-targeting treatment, and (iv) had complete headache documentation, defined as headache documentation of at least 1 month before the first injection (baseline) and at least two of three consecutive treatment cycles. Patients were excluded if they (i) switched from a CGRP ligand mAb to erenumab due to side effects, or (ii) had switched from a ligand to ligand mAb treatment before.

In our clinic, the standard care is to treat all patients for at least three consecutive months, since some patients could have a delayed treatment response. Treatment is only prematurely discontinued in case of limiting side effects.

We conducted this study according to the declaration of Helsinki. The local ethics committee approved the study (EA1/159/22). According to the national legislation and the institutional requirements, written informed consent was not required for this retrospective analysis of routinely acquired data. Our report complies with the “Strengthening the Reporting of Observational Studies in Epidemiology” (STROBE) Statement for cohort studies.

### 2.2. Definition of variables and data extraction

The number of MHD per 28 days was extracted from headache diaries. A headache day was defined as any day with documented headache. Additionally, we extracted days with acute medication use (AMD), which was defined as a headache day with use of acute medication for headache including NSAIDs, combination analgesics, and/or triptans. For missing data, we used the “Last Observation Carried Forward” approach, assuming no change. Due to the retrospective nature of this study, headache characteristics and accompanying symptoms were not always available, rendering impossible a reliable differentiation between headache days and migraine days.

The ≥30% responder rates were calculated from MHD at 3 and 6 months compared to the month before initiation of erenumab (baseline). Patients with a ≥30% decrease in MHD during month 3 were defined as responders, while patients with a < 30% decrease in MHD during month 3 were defined as non-responders.

From patient records we extracted the following patient characteristics: age, sex, diagnosis of EM or CM, age at diagnosis of migraine, disease duration, prior prophylactic treatments, and concomitant treatment with medication approved for migraine prevention (administered for migraine or a different indication, e.g., depression, epilepsy, and hypertension).

### 2.3. Endpoints

Our primary endpoint was the percentage of patients with a ≥30% reduction of MHD during month 3 of treatment with erenumab compared to baseline. Secondary endpoints were (i) the absolute reduction of MHD during month 3 compared to baseline, (ii) the absolute reduction of AMD during month 3 compared to baseline, (iii) the ≥50% reduction of MHD during month 3 of treatment with erenumab compared to baseline, and iv) the ≥30 and ≥50% reduction, absolute reduction of MHD, and absolute reduction of AMD during month 6 compared to baseline for those patients who continued treatment for at least 6 months.

### 2.4. Statistical analyses

We assessed data distribution by use of the Kolmogorov-Smirnov Test for normality. As our data were normally distributed, we used a parametric approach to test our hypothesis. To assess difference over time we estimated the statistical difference (*p*-value) and the effect size (Cohen's d for repeated measures, d_rm_). *P-*values were estimated with a Generalized Estimating Equations (GEE) statistics. A value of p ≤ 0.05 was considered statistically significant. A Cohen's d of ≥0.5 was considered as a medium effect and a Cohen's d of ≥0.8 as a large effect. Continuous variables are expressed in mean (standard deviation or 95% confidence interval), and categorical variables in n (%). Statistical analyses were performed with IBM SPSS Statistics, version 28.0.1.0 (IBM, Armonk, NY, USA).

## 3. Results

Between May 2019 and July 2022, we identified *n* = 105 and *n* = 90 patients who had received galcanezumab (240 mg loading dose followed by 120 mg monthly) and fremanezumab (225 mg monthly) as their first CGRP mAb, respectively. Patients receiving fremanezumab 675 mg in a quarterly regime were not identified since the treatment with 225 mg in a monthly regime is the preferred standard care in our clinic. Of these 195 patients, 29 (14.9%) switched to erenumab (70 mg or 140 mg monthly).

**Figure 1:**
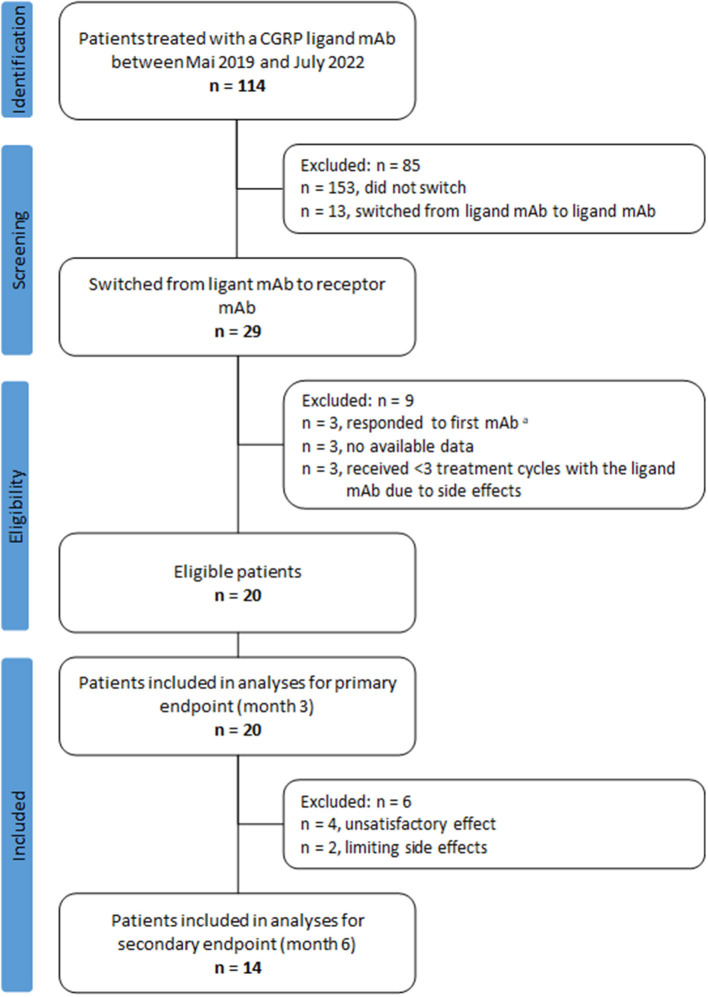
Flow chart of patient selection. ^a^Response defined as a ≥30% reduction of monthly headache days during month 3 of treatment with the CGRP ligand mAb compared to the month before treatment initiation.

We excluded nine patients: Three patients were excluded because no headache documentation was available, another three patients because they had >30% reduction in MHDs at month 3 compared to baseline during the first treatment cycle with a CGRP ligand mAb, and three patients were excluded because they received < 3 treatment cycles due to adverse events under CGRP ligand mAb treatment. This resulted in 20 patients eligible for our analyses, Figure 1.

### 3.1. Patient characteristics

Table 1 displays the patient characteristics. The mean age of our cohort was 50.7 ± 11.9 years, and 16 patients (80%) were female. Seven (35%) patients had a diagnosis of EM and 13 (65%) of CM. An average of 5.0 ± 1.4 prior prophylactic treatments had been attempted before initiation of the CGRP ligand mAb.

**Table 1:** Patient characteristics.

**Demographics**	**Total cohort**	
	***n =* 20**	
Age, years	50.7 ± 11.9	
Female	16 (80)	
Episodic migraine	7 (35)	
Chronic migraine	13 (65)	
Migraine with aura	7 (35)	
Age at onset, years	19.4 ± 15.6	
Disease duration, years	29.9 ± 15.2	
Prior prophylactic attempts	5.0 ± 1.4	
**Comorbid diseases**		
Depression	9 (45)	
Anxiety	3 (15)	
Hypertension	9 (45)	
**Prior prophylactics**	**Prior preventative**	**Contraindicated preventative**
Topiramate	17 (85)	2 (10)
Amitriptyline	16 (80)	2 (10)
Metoprolol	15 (75)	1 (5)
BotulinumtoxinA	13 (65)	0 (0)
Flunarizine	12 (60)	8 (40)
Venlafaxine	6 (30)	0 (0)
Propranolol	5 (25)	1 (5)
Candesartan	4 (20)	0 (0)
Bisoprolol	3 (15)	1 (5)
Mirtazapine	2 (10)	0 (0)
Valproate	2 (10)	0 (0)
Other	4 (20)	0 (0)

Values are given in mean ± SD or *n* (%).

### 3.2. Treatment with a CGRP ligand mAb

Fourteen patients (70%) were treated with galcanezumab and six patients (30%) were treated with fremanezumab. Patients treated with galcanezumab received 5.9 ± 3.0 administrations and patients treated with fremanezumab received 5.5 ± 2.9 administrations prior to switching, respectively (*p* = 0.405).

### 3.3. Break between CGRP mAb

After termination of the CGRP ligand mAb after a minimum of three administrations, patients had a mean break of 129.7 ± 46.2 (range: 75–279) days [137.2 ± 50.0 (range: 91–279) for galcanezumab and 112.0 ± 33.0 (range: 75–279) for fremanezumab], before beginning erenumab.

### 3.4. Treatment with a CGRP receptor mAb

From 20 patients who switched to erenumab, the dose of 70 mg erenumab was given to 18 (90%) patients; the other two patients started with 140 mg erenumab. For nine (45%) patients the dosage was not increased during the first three treatment cycles. An increase from 70 to 140 mg erenumab occurred in four patients during the second cycle and in five patients during the third cycle. None of the patients discontinued treatment during the first 3 months. Six patients (30%) discontinued treatment before month 6 (unsatisfactory effect, *n* = 4 [20%] and limiting side effects, *n* = 2 [10%]).

### 3.5. Concomitant treatments

None of the patients received concomitant migraine prophylaxis. Nevertheless, eight patients (40%) received concomitant treatment for a comorbid disease. All concurrent treatments were administered at a stable dose and remained unchanged throughout the full observation period including treatment with both CGRP mAbs. Table 2 gives an overview of the concomitant treatments and their indications.

**Table 2:** Concomitant treatments.

**Patient**	**Concomitant treatment**	**Indication**
Patient 1	Candesartan 16 mg	Hypertension
Patient 2	Candesartan 16 mg	Hypertension
Patient 3	Duloxetine 90 mg	Depression
Patient 4	Lamotrigine 150 mg	Depression
Patient 5	Metoprolol 95 mg	Hypertension
Patient 6	Ramipril 2.5 mg	Hypertension
Patient 7	Bisoprolol 25 mg	Hypertension
	Venlafaxine 150 mg	Depression
Patient 8	Amitriptyline 10 mg	Depression
	Metoprolol 47.5 mg	Hypertension
	Ramipril 2.5 mg	Hypertension

### 3.6. Monthly headache days and responder rates

Due to the definition of our inclusion criteria, none of our patients had a ≥30% reduction of MHD after 3 administrations of a CGRP ligand mAb compared to the month before initiation of the CGRP ligand mAb. MHD changed from 17.9 ± 7.0 days during the month before initiation of the CGRP ligand mAb to 17.8 ± 7.0 days during month 3 of treatment, *p* = 0.769, d_rm_ = 0.022 (Supplementary Table 1). During the month before initiation of erenumab, the mean MHDs were 18.6 ± 5.9 days and decreased significantly to 14.5 ± 5.9 days during month 3 of treatment, *p* < 0.001, d_rm_ = 0.747 (Table 3). At 6 months of treatment, in the remaining 14 patients MHD further decreased to 11.6 ± 4.6 days, *p* < 0.001, d_rm_ = 1.146.

**Table 3:** Change of monthly headache days after switch to receptor antibody.

		**Baseline**	**Change from baseline**		
			**Month 1**	**Month 3**	**Month 6 (*n =* 14)**
	***n***	**Mean (95% CI)**	**Mean (95% CI)**	**Mean (95% CI)**	**Mean (95% CI)**
**Monthly headache days**	20	18.6 (16.0 to 21.1)	−2.8 (−4.1 to −1.5)	−4.1 (−5.6 to −2.5)	−7.0 (−9.1 to −4.9)
**Monthly acute medication days**	17	11.6 (8.8 to 14.4)	−2.5 (−4.8 to −0.2)	−3.4 (−5.6 to −1.0)	−2.6 (−6.1 to 0.9)

^*^Statistically significant *p* < 0.05.

**Figure 2:**
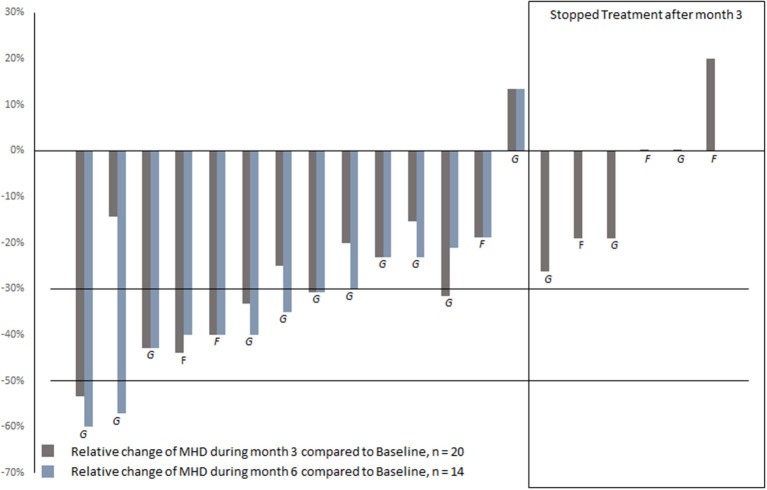
Reduction of MHD during month 3 and 6 of treatment with erenumab, compared to baseline. G, galcanezumab as prior CGRP ligand mAb; F, fremanezumab as prior CGRP ligand mAb.

A reduction of ≥30% was achieved by seven patients (35%) during month 3 of treatment with erenumab compared to baseline, including one patient (5%) with a reduction of ≥50%, Figure 2. During month 6, nine (45%) patients achieved a ≥30% reduction of MHD compared to baseline, including two patients (10%) with a reduction of ≥50%. Responders did not differ from non-responders with regard to age, sex, migraine diagnosis [EM, CM], age at migraine onset, migraine disease duration, comorbidities [depression, anxiety, and hypertension], the type of ligand mAb before initiation of erenumab, MHD before initiation of CGRP ligand/receptor mAb treatment, the duration of the break between treatments, and the treatment dose of erenumab [70 mg, 140 mg] (Supplementary Table 2).

### 3.7. Monthly acute medication days

AMD decreased from 11.6 ± 6.1 days during the month before initiation of erenumab to 8.2 ± 4.8 days during month 3 of treatment, *p* = 0.006 (Table 3). During month 6, data from 12 patients were available. Patients reported 9.0 ± 4.5 AMD which was not significantly different from baseline *p* = 0.147, d_rm_ = 0.618.

### 3.8. Daily and near-daily headache

Five patients (25%) reported headache on ≥25/28 days during the entire treatment with the CGRP ligand mAb, and during the month before initiation of erenumab. From these, three patients reported daily headache (28/28 days). At 3 months of treatment with erenumab, MHD decreased to ≤ 20 days in 3/5 patients (60%) with sustained treatment efficacy during month 6. Two patients with daily headache continued to report headache on 28/28 days during month 3 and discontinued treatment after 3 months.

## 4. Discussion

In this retrospective real-world study, switching to the CGRP receptor mAb erenumab resulted in a ≥30% reduction of MHD at 3 months in 35% of patients who had not responded to CGRP ligand mAbs previously. At 6 months, the ≥30% responder rate increased to 45%. Mean MHD decreased by 4.1 days at 3 months and by 7.0 days at 6 months compared to baseline, respectively.

Erenumab has been authorized by the European Medicines Agency in 2018, and the CGRP ligand mAbs fremanezumab and galcanezumab in 2019 (26). Thus, previous European real-world studies on switching between CGRP mAbs have mainly addressed a switch from erenumab to CGRP ligand mAbs. A prior study from our group on treatment efficacy after switching to CGRP ligand mAbs in 25 non-responders to erenumab found a ≥30% reduction of MHD in 32% of patients at 3 months (16). The efficacy at 6 months was not reported. In our present study, the effect of erenumab after failure of a CGRP ligand mAb seems to be comparable to the efficacy of a switch in the opposite direction. The largest analysis on switching between CGRP mAbs has been performed within the real-world multicenter study FINESSE which assesses effectiveness and tolerability of fremanezumab in migraine patients with or without prior CGRP-targeting treatment (27). According to preliminary and not yet peer-reviewed data, from 57 patients who switched to fremanezumab due to lack of efficacy of either galcanezumab or erenumab, 32% reached the primary endpoint of a ≥50% reduction of MMD (19). The ≥30% response rate was not reported. Possibly, the higher response rate compared to our study might be explained by a lower percentage of patients with CM in FINESSE compared to our cohort (43.8 vs. 65%). Indeed, the absolute reduction of MMD at 6 months was comparable to the reduction of MHD in our study with 5.3 days in the overall cohort, 4.6 days in patients with EM and 6.7 days in patients with CM for FINESSE. Nevertheless, the assessment of MMD in FINESSE vs. MHD in our study limits the comparability of both studies.

One real-world study on switching from a CGRP ligand mAb to erenumab in 22 patients reported neither the number of previous treatments, nor the number of absolute MHD/MMD at baseline and follow-up, nor the number of AMD (20). Thus, a comparison with our cohort is limited. Other results on switching between CGRP mAbs have been published in conference posters without available data on the direction of the switch (e.g., CGRP ligand mAb to receptor or vice versa) (28, 29).

Up to now, no study has directly compared the efficacy of different CGRP mAbs. Although CGRP ligand mAbs and CGRP receptor mAbs have different modes of action, an indirect comparison between responder rates from the respective RCT and RWS does not indicate superiority of one CGRP mAb over another (2, 3). To date, it remains unclear why some patients respond to a CGRP receptor mAb but not to a CGRP ligand mAb and vice versa. CGRP belongs to the calcitonin family of peptides, including α-CGRP and β-CGRP, calcitonin, amylin, adrenomedullin, and adrenomedullin 2, and pharmacological relationships between these different peptides of the calcitonin family and their receptors are complex (30). Experimental data has shown that CGRP does not only bind to the CGRP receptor, but also to receptors for amylin, calcitonin, and adrenomedullin (31, 32). On the other hand, application of adrenomedullin and amylin may activate the CGRP receptor and has been shown to induce migraine-like headaches in migraine patients (30, 33). Thus, it is conceivable that CGRP receptor mAb and CGRP ligand mAb modulate several non-CGRP pathways to various degrees in different patients, resulting in inter-individual differences in response to CGRP mAb treatment.

Our cohort is characterized by a predominance of CM, a long disease duration, a high number of prior treatment failures, and a high prevalence of comorbid depression. This differs from the population of RCT mostly including strictly selected patients but is representative of RWS populations (34). The high number of prior treatment failures is attributable to reimbursement criteria in Germany, as at the time of recruitment, several prior treatment failures were required to gain access to CGRP mAbs. We describe a difficult-to-treat population with all patients fulfilling criteria for resistant migraine, defined by having failed at least 3 classes of migraine preventatives and suffering from at least 8 debilitating headache days per month for at least 3 consecutive months (35). Therefore, we chose a ≥30% reduction of MHD instead of a ≥50% reduction as the primary outcome. In this population of difficult-to-treat patients, a reduction of ≥30% can signify a major improvement for the patient. In patients who continued erenumab treatment during month 6, the mean absolute number of MHD was reduced by 7 days. While there is no data on treatment efficacy of erenumab in patients with prior non-response to CGRP ligand mAbs, real-world studies evaluating treatment efficacy of erenumab as a first CGRP mAb have found a comparable absolute reduction of MHD at 6 months for patients who had failed 8.4 ± 3.6 previous preventive treatments (13), and a higher absolute reduction of 12.2 MHD and 15 MMD in patients who had failed 4.7 ± 0.3 and ≥2 prior preventative treatments, respectively (12, 14).

In contrast to the number of MHD, the number of AMD decreased significantly from baseline to month 3, but not to month 6 of treatment with erenumab. However, interpretation of these data is limited by a lack of power, as data were available from only 12 patients.

Of note, the classification of patients as responders or non-responders dependent on the reduction of MHD is a one-dimensional approach that might not always reflect the actual benefit as perceived by the patient. When taking into account other outcome measures such as pain severity and headache impact, some patients benefit from CGRP mAbs although being classified as non-responders (36, 37). In our clinic, outcome measures beyond MHD are not systematically documented. Since this is a real world-study with retrospective data acquisition, we were not able to include other relevant outcomes such as quality of life or patients' global impression of change. Further prospective studies on switching between CGRP mAbs in non-responders should choose a multi-dimensional outcome evaluation and include those patient-reported outcome parameters in addition to the reduction of MHD to acquire a more comprehensive assessment of treatment efficacy in this population (38).

One strength of this study is the follow-up period of 6 instead of 3 months, exceeding the follow up-period reported in previously published studies on switching between CGRP mAbs (16–18). The percentage of ≥30% responders increased from month 3 to month 6, which is valuable additional information favoring a longer period of observation after a switch. Trials on first initiation of CGRP mAbs treatment have shown that while in most responders the treatment effect is achieved quickly and within the first 3 months of treatment, responder rates increase over time (39, 40). In line with these results, real-world studies evaluating efficacy of erenumab at 3 and 6 months reported a higher response rate at 6 compared to 3 months of treatment (12–14). Consequently, the EHF guidelines recommend the first evaluation of CGRP mAb treatment efficacy after a minimum of 3 months, and in selected cases a re-evaluation after an additional period of 3 months (41). The identification of predictors for the time course and durability of response could allow for more individualized recommendations.

In clinical practice, one of the most relevant questions is how to predict which patients will benefit from switching between CGRP mAbs. Due to the small number of patients, we were not able to identify differences between responders and non-responders in this cohort. Our previous study on switching from a CGRP ligand mAb to erenumab revealed that patients with daily headache were less likely to benefit from a switch (16). In this cohort, the smaller number of patients with daily or near-daily headache did not allow for a stratified analysis, but 3/5 patients reported a reduction of MHD and continued treatment for ≥6 months. Other studies addressing differences between responders and non-responders to a first treatment with a CGRP mAb found an association of psychiatric comorbidities, a long disease duration, and a high number of previously failed preventive treatments with a lower response (14, 42, 43). Future larger multi-centric studies are necessary to describe clinical predictors for the efficacy of switching between CGRP mAbs.

For all included patients, headache diaries were available as source data and were carefully reviewed for the number of MHD. However, sometimes patients were not able to differentiate between MHD (including migraine days) and MMD, or the differentiation was not clearly documented. Therefore, we documented MHD instead of MMD, which might restrict comparability with other studies that examined MMD. Nevertheless, MHD seems to be a suitable outcome parameter since they correlate with disability in migraine patients (44).

Further limitations of our study include the retrospective non-blinded design and the absence of a control group, possibly introducing bias and placebo response. Several studies have demonstrated that in pain disorders including migraine and osteoarthritic pain, sham injections (subcutaneous or intra-articular, respectively) may have a prolonged placebo effect of up to 6 months (45, 46). Therefore, we cannot exclude a potential role of a placebo effect in our findings. Our cohort is representative of real-world studies, but especially due to the small sample size, our findings might not be generalizable to all migraine patients with non-response to a CGRP ligand mAb. Nine patients (45%) received the lower dose of erenumab 70 mg during the entire course of observation, and results might vary in a cohort of patients treated with 140 mg. Due to the small sample size, we were not able to determine if a switch from galcanezumab to erenumab and from fremanezumab to erenumab was equally effective.

To conclude, our data suggest that switching from a CGRP ligand mAb to erenumab might be beneficial in a subgroup of resistant patients and that the responder rates might increase after 6 months of treatment. A switch could therefore be a justifiable measure for non-responders, especially when alternatives are scarce. Besides these considerations on efficacy, the beneficial safety profile and the good tolerability of CGRP mAbs are in favor of a switch as a reasonable treatment attempt. Larger prospective studies should aim to predict which subgroups of patients benefit from a switch.

## Data availability statement

The raw data supporting the conclusions of this article will be made available upon reasonable request.

## Ethics statement

The studies involving human participants were reviewed and approved by Charité Ethical Committee (EA1/159/22). Written informed consent for participation was not required for this study in accordance with the national legislation and the institutional requirements.

## Author contributions

LO, KL, and BR designed the study and interpreted the data. LO, KL, MF, AS, MS, PT, and BR contributed to data collection. LO and KL analyzed the data and wrote the first draft of the manuscript. MF, AS, MS, PT, JH, and UR critically reviewed the article for important intellectual content. All authors read and approved the final manuscript.
