# Supplementary material for: Effect of switching to erenumab in non-responders to a CGRP ligand antibody treatment in migraine: A real-world cohort study
Source: Front Neurol. 2023 Mar 22;14:1154420. doi: 10.3389/fneur.2023.1154420 (PMC10075077; doi:10.3389/fneur.2023.1154420)
Supplement: Supplementary file 1 [file Table_1.docx]

Effect of switching to erenumab in non-responders to a CGRP ligand antibody treatment in migraine: A real-world cohort study

**SUPPLEMENTARY MATERIAL**

| **Sup.Table 1** Change of monthly headache days during the first three months under treatment with a CGRP ligand mAb | | | | |
| --- | --- | --- | --- | --- |
|  |  | **Baseline** | **Change from baseline** | |
|  |  |  | **Month 1** | **Month 3** |
|  | **n** | **Mean**  **(95% CI)** | **Mean**  **(95% CI)** | **Mean**  **(95% CI)** |
| **Monthly**  **headache days** | 20 | 17.9 | -0.3 | -0.2 |
|  |  | (14.6 to 21.2) | (-1.1 to 0.6)  p = 0.555  d_rm_ = 0.036 | (-1.2 to 0.9)  p = 0.769  d_rm_ = 0.022 |
| * Statistically significant p < 0.05. | | | | |

| **Sup.Table 2** Comparison of characteristic of non-responder vs responder during month 3 and month 6 | | | | | | |
| --- | --- | --- | --- | --- | --- | --- |
|  | **Non-responder Month 3 (n = 13)** | **Responder Month 3 (n = 7)** | **p-value** | **Non-responder Month 6 (n = 11)** | **Responder Month 6 (n = 9)** | **p-value** |
| Female | 11 (84.6) | 5 (71.4) | 0.587 | 10 (90.9) | 6 (66.7) | 0.285 |
| Use of concomitant drugs | 5 (38.5) | 3 (42.9) | 1.000 | 4 (36.4) | 4 (44.4) | 1.000 |
| Episodic migraine | 3 (23.1) | 4 (57.1) | 0.174 | 2 (18.2) | 5 (55.6) | 0.160 |
| Chronic migraine | 10 (76.9) | 3 (42.9) |  | 9 (81.8) | 4 (44.4) |  |
| Migraine with aura | 3 (23.1) | 4 (57.1) | 0.174 | 2 (18.2) | 5 (55.6) | 0.160 |
| Depression | 4 (30.8) | 5 (71.4) | 0.160 | 3 (27.3) | 6 (66.7) | 0.175 |
| Anxiety | 1 (7.7) | 2 (28.6) | 0.521 | 0 (0) | 3 (33.3) | 0.074 |
| Hypertension | 6 (46.2) | 3 (42.9) | 1.000 | 5 (45.5) | 4 (44.4) | 1.000 |
| Priorly Galcanezumab | 9 (45.0) | 5 (25.0) | 1.000 | 7 (35.0) | 7 (35.0) | 0.642 |
| Priorly Fremanezumab | 4 (20.0) | 2 (10.0) |  | 4 (20.0) | 2 (10.0) |  |
| Disease duration, years | 33.1 ± 14.1 | 22.2 ± 16.7 | 0.315 | 33.7 ± 14.4 | 22.8 ± 15.4 | 0.171 |
| Age at onset, years | 18.5 ± 16.8 | 21.6 ± 13.6 | 0.556 | 19 ± 17.7 | 20.2 ± 12.3 | 0.608 |
| Age, years | 52.1 ± 11.6 | 48.1 ± 12.9 | 0.438 | 52.8 ± 11.4 | 48.1 ± 12.7 | 0.456 |
| Number of treatment cycles with the first mAb | 6.2 ± 3 | 4.9 ± 2.9 | 0.281 | 5.4 ± 2.3 | 6.2 ± 3.7 | 0.743 |
| MHDs before CGRP ligand mAb treatment | 17.9 ± 7.3 | 17.9 ± 7.1 | 0.987 | 19 ± 6.9 | 16.6 ± 7.4 | 0.465 |
| MHDs before CGRP-receptor mAb treatment | 18.9 ± 5.8 | 17.9 ± 6.5 | 0.600 | 20.1 ± 5.3 | 16.7 ± 6.4 | 0.133 |
| Prior prophylactic attempts | 5.2 ± 1.4 | 4.4 ± 1.3 | 0.251 | 5.3 ± 1.3 | 4.6 ± 1.4 | 0.398 |
| Break duration | 4.9 ± 2 | 4 ± 0.8 | 0.393 | 5 ± 2.1 | 4 ± 0.7 | 0.295 |
| Dose increase of CGRP-receptor mAb | 2.4 ± 1.5 | 3 ± 3 | 0.891 | 2.5 ± 1.4 | 2.7 ± 2.8 | 0.752 |
